# Role of the Mitochondrial Pyruvate Carrier in the Occurrence of Metabolic Inflexibility in *Drosophila melanogaster* Exposed to Dietary Sucrose

**DOI:** 10.3390/metabo10100411

**Published:** 2020-10-14

**Authors:** Chloé J. Simard, Mohamed Touaibia, Eric Pierre Allain, Etienne Hebert-Chatelain, Nicolas Pichaud

**Affiliations:** 1 Department of Chemistry and Biochemistry, Université de Moncton, Moncton, NB E1A 3E9, Canada; ecs0175@umoncton.ca (C.J.S.); mohamed.touaibia@umoncton.ca (M.T.); 2 Atlantic Cancer Research Institute (ACRI), Moncton, NB E1C 8x3, Canada; erica@canceratl.ca; 3 New Brunswick Centre for Precision Medicine (NBCPM), Moncton, NB E1A 3E9, Canada; etienne.hebert.chatelain@umoncton.ca; 4 Department of Biology, Université de Moncton, Moncton, NB E1A 3E9, Canada; 5 Canada Research Chair in Mitochondrial Signaling and Physiopathology, Moncton, NB E1A 3E9, Canada

**Keywords:** Drosophila, mitochondrial pyruvate carrier, mitochondrial respiration, homeostasis, sucrose, metabolomics, metabolic inflexibility

## Abstract

Excess dietary carbohydrates are linked to dysregulation of metabolic pathways converging to mitochondria and metabolic inflexibility. Here, we determined the role of the mitochondrial pyruvate carrier (MPC) in the occurrence of this metabolic inflexibility in wild-type (WT) and MPC1-deficient (MPC1^def^) flies that were exposed to diets with different sucrose concentrations for 15–25 days (Standard Diet: SD, Medium-Sucrose Diet: MSD, and High-Sucrose Diet: HSD). Our results showed that MPC1^def^ flies had lower mitochondrial respiration rates than WT flies on the SD and MSD. However, when exposed to the HSD, WT flies displayed decreased mitochondrial respiration rates compared to MPC1^def^ flies. WT flies exposed to the HSD also displayed increased proline contribution and slightly decreased *MPC1* expression. Surprisingly, when fed the MSD and the HSD, few metabolites were altered in WT flies whereas MPC1^def^ flies display significant accumulation of glycogen, glucose, fructose, lactate, and glycerol. Overall, this suggests that metabolic inflexibility starts to occur in WT flies after 15–25 days of exposure to the HSD whereas the MPC1^def^ flies display metabolic inflexibility independently of the diet provided. This study thus highlights the involvement of MPC as an essential protein in Drosophila to maintain proper metabolic homeostasis during changes in dietary resources.

## 1. Introduction

A change in dietary resources is an important environmental parameter for organisms, as it can be a major driving force affecting molecular, metabolic, and physiological aspects of phenotype. As such, key components of a fine-tuned metabolic network have to properly respond to local and systemic nutrient environments to maintain cell homeostasis [1,2,3]. However, inadequate adjustments to nutritional cues can lead to several metabolic dysfunctions. Recently, the involvement of carbohydrate overconsumption in the development of metabolic disorders has been linked to the dysregulation of the different metabolic pathways converging to mitochondria [2,4,5,6,7]. Mitochondria are at the center of intermediary metabolism; they not only produce most of the ATP required for the proper functioning of the cell but also are the hub for the tricarboxylic acid cycle (TCA cycle), the electron transport system (ETS), the fatty acid β-oxidation as well as for protein catabolism and amino acid interconversion (among other important metabolic pathways).

During normal physiological conditions, different fuels can be alternatively oxidized by mitochondria according to physiological and nutritional circumstances. Carbohydrates such as glucose are first processed by the glycolysis in the cytosol, which produces pyruvate. Pyruvate can then be transported inside the mitochondria to be further metabolized into acetyl-CoA. Oxidation of fatty acids and of several amino acids also yields this acetyl-CoA, that will then enter the TCA cycle, producing the reducing equivalents nicotinamide adenine dinucleotide (NADH) and flavin adenine dinucleotide (FADH_2_). The ETS protein complexes (complexes I to IV) embedded in the mitochondrial inner membrane then use these reducing equivalents as electron donors, which are transferred from one complex to the other, until the final acceptor of the system, dioxygen. This electron transport generates a proton gradient across the inner mitochondrial membrane that is then used to fuel the ATP synthase (complex V) to produce ATP by the oxidative phosphorylation (OXPHOS) process. In addition to the classic ETS complexes, several amino acids, fatty acids, as well as other metabolites can be transformed by alternative complexes of the ETS, such as proline dehydrogenase (ProDH) or mitochondrial glycerol-3-phosphate dehydrogenase (mG3PDH) to increase the flux of electrons inside the ETS [8,9,10].

However, when excess substrates derived from the dietary macronutrients are present, metabolic inflexibility can occur; the cell becomes overwhelmed in the presence of excess nutrient and loses the capacity to self-regulate its metabolism correctly, leading to an incapacity of mitochondria to switch between oxidative substrates [2]. Thus, it has been suggested that alleviating mitochondrial substrate competition by blocking one of the pathways leading to the influx of carbons inside the mitochondria could prevent and/or mitigate the development of metabolic disorders [2,11,12,13]. Amongst all the different pathways and metabolites necessary for the proper functioning of mitochondrial metabolism, pyruvate is a key molecule [14,15,16,17]. First, it is central to glucose metabolism and homeostasis as it is the end-product of glycolysis. As such, it is also a critical intermediate of aerobic and anaerobic metabolism, gluconeogenesis, the TCA cycle, and amino acid metabolism. Pyruvate alone is unable to diffuse through the inner mitochondrial membrane from the cytosol. A specialized protein embedded in the inner mitochondrial membrane, the mitochondrial pyruvate carrier (MPC), is responsible for this transport. This protein is widely conserved and has been identified in different species, from yeast to humans [16,18]. Deletion of one of the two proteins constituting the MPC (MPC1 and MPC2) results in elevated blood sugar concentrations and glucose intolerance accompanied by impaired glucose-stimulated insulin secretion in both Drosophila and mice [16]. However, inhibition of MPC through pharmacological and genomic approaches has also resulted in the reduction of several metabolic impairments [15,16,19,20]. For example, it has been shown that mice harboring a loss of MPC2 expression in their hepatocytes possessed a limited pyruvate contribution to gluconeogenesis, leading to reduced glucose production and thus decreasing glycemia [15]. Similarly, constitutive liver-specific deletion of MPC1 attenuates the development of hyperglycemia induced by a high-fat diet in mice [21]. However, it is unclear whether and how blocking the pyruvate influx into the mitochondria could alleviate mitochondrial substrate competition and metabolic inflexibility during nutrient excess.

In this study, we evaluated the effect of dietary sucrose on the intermediary metabolism of Drosophila harboring a mild MPC1 deficiency (MPC1^def^ flies). We previously demonstrated that the MPC1^def^ adult flies fed a standard diet (SD) display a general reduction of metabolic functions, with significantly reduced climbing capacity, pyruvate-induced oxygen consumption, enzymatic activities of pyruvate kinase, alanine aminotransferase, and citrate synthase compared to wild-type (WT) flies [22]. Moreover, increased proline oxidation capacity was detected in MPC1^def^ flies, which was associated with generally lower levels of several metabolites, particularly those involved in amino acid catabolism [22]. We concluded that MPC is an important metabolic control point for mitochondrial substrate oxidation, as a mild deficiency of pyruvate import can reshape the mitochondrial metabolism to favor amino acids instead of carbohydrates as oxidative substrates [22]. Here, we used a comparative approach to determine the role of MPC in mitochondrial substrate oxidation and metabolism when WT and MPC1^def^ flies were exposed to diets with different sucrose concentrations. Specifically, we measured mitochondrial respiration at several steps of the ETS using high-resolution respirometry and metabolite levels using ^1^H nuclear magnetic resonance (NMR) spectroscopy in WT and MPC1^def^ flies fed the SD, a medium-sucrose diet (MSD: 18% of dietary sucrose), and a high-sucrose diet (HSD: 30% of dietary sucrose). We hypothesized that, when exposed to high content of carbohydrates in their diet, flies exhibiting MPC1 deficiency will have an advantage over WT flies to use amino acids as oxidative substrates, which will result in reduced mitochondrial substrate competition and metabolic inflexibility compared to WT flies.

## 2. Results

Drosophila melanogaster genotypes w^1118^ and w[1118]; P{w[+mC] = XP}Mpc1[d00809], thereafter referred as WT and MPC1^def^, were raised on a SD until they reach 15 days old. Each genotype was then transferred to the SD, the MSD (18% of dietary sucrose), or the HSD (30% of dietary sucrose) for an additional 15–25 days before being collected at 30–40 days old for experiments.

### 2.1. MPC1 Gene Expression

The MPC1^def^ genotype was generated using P-element insertion which frequently interferes directly with gene function [23,24,25] and was previously characterized by our group at the mitochondrial level (see [22]). *MPC1* gene expression was highly influenced by the genotype (F_1,24_ = 17.27, *p* < 0.001) but not by the diet or by the interaction genotype × diet. As expected, MPC1^def^ flies presented overall reduced *MPC1* gene expression than WT flies. Specifically, relative *MPC1* gene expression was reduced in MPC1^def^ flies fed the SD the MSD; and to a lesser extent, the HSD (Table 1).

Interestingly, when gene expression was normalized relatively to WT flies fed the SD, a slight decrease was observed in relative *MPC1* expression according to the diet in WT flies as well as in MPC1^def^ flies (Table 1).

### 2.2. Mitochondrial Respiration

#### 2.2.1. Mitochondrial Respiration Rates

We measured mitochondrial oxygen consumption with the addition of various substrates to observe different steps of the respiration process (see the Materials and Methods section for details) according to [26]. All F-values obtained following two-way ANOVAs for mitochondrial respiration rates and mitochondrial ratios are presented in the Supplementary Materials (Table S1).

**Figure 1:**
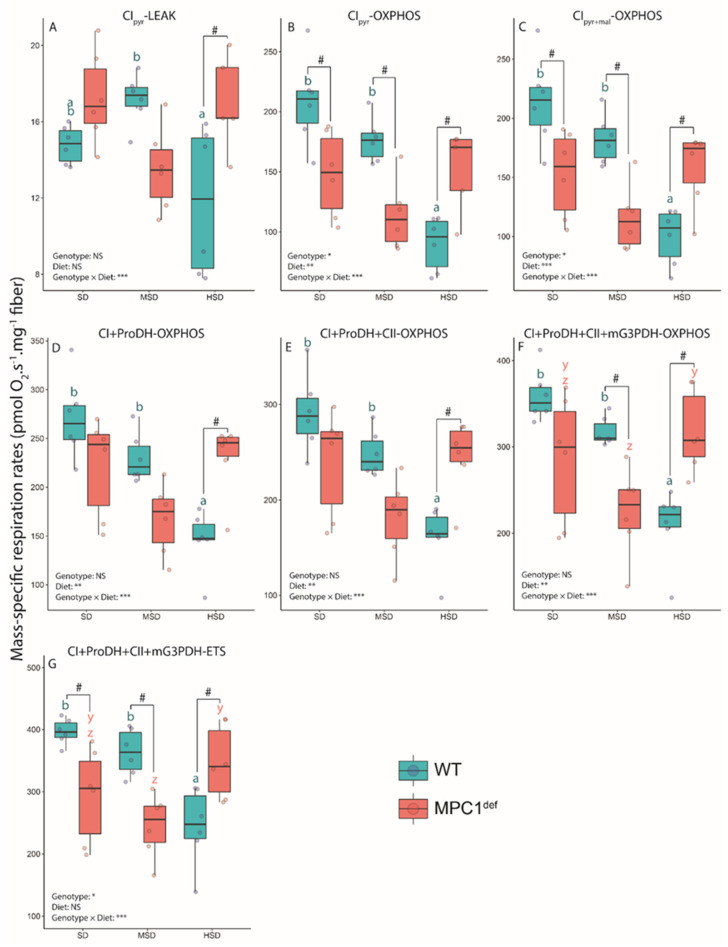
Mass-specific mitochondrial oxygen consumption rates measured in permeabilized thorax of *Drosophila melanogaster* WT and MPC1^def^ exposed to the different experimental diets (SD, MSD, or HSD): (**A**) pyruvate (CI_pyr_-LEAK), (**B**) +ADP (CI_pyr_-OXPHOS), (**C**) +malate (CI_pyr+mal_-OXPHOS), (**D**) +proline (CI+ProDH-OXPHOS), (**E**) +succinate (CI+ProDH+CII-OXPHOS), (**F**) +G3P (CI+ProDH+CII+mG3PDH-OXPHOS), and (**G**) +FCCP (CI+ProDH+CII+mG3PDH-ETS). The results correspond to the mean ± SEM for each condition (N = 6). Statistical analysis: stars represent statistical results from ANOVAs with * *p* < 0.05, ** *p* < 0.01, and *** *p* < 0.001; # represents significant differences between genotypes for each experimental diet detected using the estimated marginal means method; and letters denote differences between experimental diets for each genotype, with “a” and “b” representing differences between the WT flies and “y” and “z” representing differences between the MPC1^def^ flies.

For the CI_pyr_-LEAK respiration, we found a significantly higher respiration rate in the MPC1^def^ flies compared to the WT flies but only when they were fed the HSD (*p* = 0.024; Figure 1A). In the WT flies, the CI_pyr_-LEAK respiration was also significantly decreased when the flies were fed the HSD compared to those fed the MSD (*p* = 0.013; Figure 1A).

We then measured the OXPHOS respiration with the addition of ADP and several other substrates (Figure 1B–F). We observed a significant decrease of oxygen consumption in WT flies fed the HSD compared to those fed the SD and MSD after addition of ADP with or without malate (CI_pyr_-OXPHOS and CI_pyr+mal_-OXPHOS; all *p*-values < 0.001; Figure 1B,C). No significant differences were observed among MPC1^def^ flies fed the different diets. Generally, WT flies had significantly higher respiration rates than MPC1^def^ flies exposed to the SD and MSD (*p* = 0.017 and *p* = 0.013 for CI_pyr_-OXPHOS and *p* = 0.017 and *p* = 0.008 for CI_pyr+mal_-OXPHOS, respectively; Figure 1B,C). For the HSD, the opposite was observed, with the WT flies having lower rates than the MPC1^def^ flies for CI_pyr_-OXPHOS (*p* = 0.024; Figure 1B) and CI_pyr+mal_-OXPHOS (*p* = 0.027; Figure 1C).

With the addition of proline (CI+ProDH-OXPHOS, Figure 1D) and succinate (CI+ProDH+CII-OXPHOS, Figure 1E), we observed decreased oxygen consumption when the WT flies were fed the HSD compared to those fed the SD and MSD (*p* < 0.001 and *p* = 0.007 for CI+ProDH-OXPHOS, respectively; *p* < 0.001 and *p* = 0.009 for CI+ProDH+CII-OXPHOS, respectively; Figure 1D,E). However, similar respiration rates were measured for the MPC1^def^ flies across diets. Significant differences between WT and MPC1^def^ flies were also detected when fed the HSD (*p* = 0.007 for CI+ProDH-OXPHOS and *p* = 0.013 for CI+ProDH+CII-OXPHOS; Figure 1D,E). Upon addition of G3P (CI+ProDH+CII+mG3PDH-OXPHOS; Figure 1F), WT flies fed the HSD had lower respiration rates than those fed the SD or MSD (*p* < 0.001 and *p* = 0.005, respectively). Moreover, the MPC1^def^ flies fed the HSD had higher respiration rates than those fed the MSD (*p* = 0.021; Figure 1F), but no differences were observed compared to SD. Significant differences between WT and MPC1^def^ flies fed the MSD and the HSD were also observed (*p* = 0.021 and *p* = 0.005; Figure 1F).

We then measured the ETS capacity which is the maximal respiration rate achieved by adding carbonyl cyanide-p-trifluoromethoxyphenylhydrazone (FCCP), representing the non-coupled respiration (CI+ProDH+CII+mG3PDH-ETS; Figure 1G). Again, higher respiration rates were observed in the WT flies fed the SD and the MSD compared to the HSD (*p* < 0.001 and *p* = 0.007, respectively; Figure 1G) as well as for the MPC1^def^ flies fed the HSD compared to the MSD (*p* = 0.029; Figure 1G). Moreover, significantly higher respiration rates were detected in WT flies fed the SD and the MSD compared to the MPC1^def^ flies (*p* = 0.027 and *p* = 0.008, respectively; Figure 1G), but decreased respiration rates in WT flies fed the HSD compared to the MPC1^def^ flies fed the same diet were detected (*p* = 0.027; Figure 1G).

#### 2.2.2. Mitochondrial Ratios

**Figure 2:**
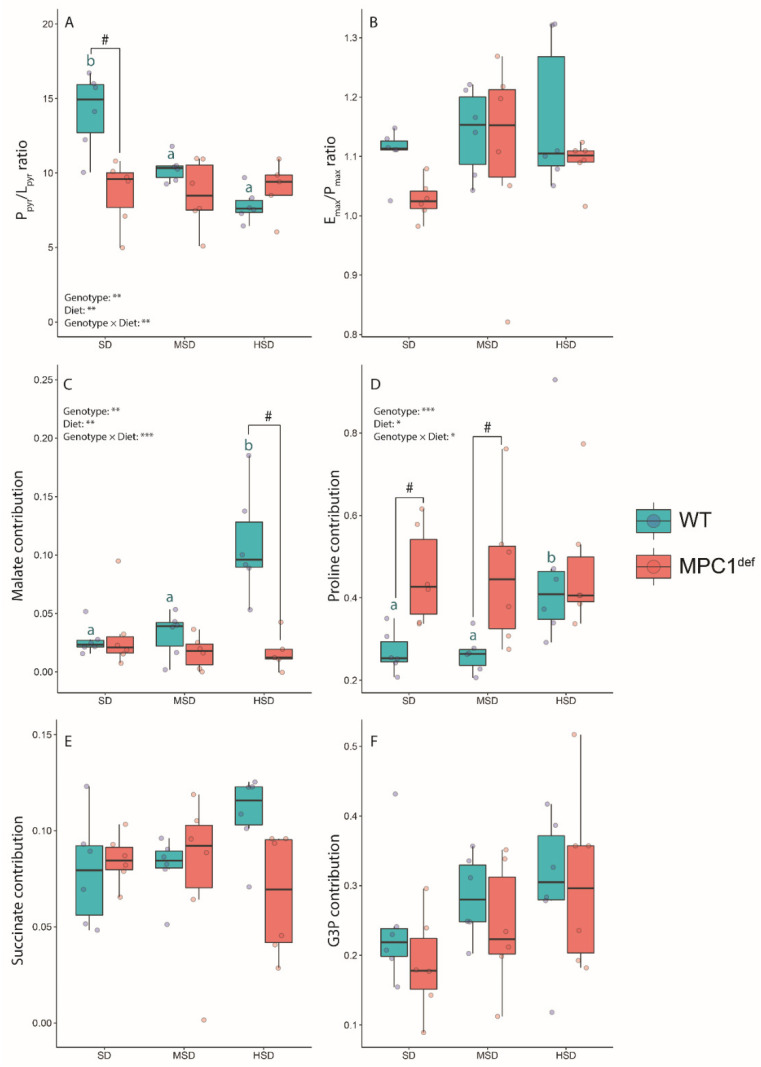
Calculated mitochondrial ratios and substrate contributions measured in permeabilized thorax of *Drosophila melanogaster* WT and MPC1^def^ exposed to the different experimental diets (SD, MSD, or HSD): (**A**) P_pyr_/L_pyr_ ratio (CI_pyr_-OXPHOS/CI_pyr_-LEAK), (**B**) E_max_/P_max_ ratio (CI+ProDH+CII+mG3PDH-ETS/CI+ProDH+CII+mG3PDH-OXPHOS), (**C**) malate contribution ((CI_pyr+mal_-OXPHOS − CI_pyr_-OXPHOS)/CI_pyr_-OXPHOS), (**D**) proline contribution ((CI+ProDH-OXPHOS − CIc-OXPHOS)/CIc-OXPHOS), (**E**) succinate contribution ((CI+ProDH+CII-OXPHOS − CI+ProDH-OXPHOS)/CI+ProDH-OXPHOS), and (**F**) G3P contribution ((CI+ProDH+CII+mG3PDH-OXPHOS − CI+ProDH+CII-OXPHOS)/CI+ProDH+CII-OXPHOS). The results correspond to the mean ± SEM for each condition (N = 6). Statistical analysis: stars represent statistical results from ANOVAs with * *p* < 0.05, ** *p* < 0.01, and *** *p* < 0.001; # represents significant differences between genotypes for each experimental diet detected using the estimated marginal means method; and letters denote differences between experimental diets for each genotype, with “a” and “b” representing differences between the WT flies.

Different mitochondrial ratios were calculated from the respiration rates. The P_pyr_/L_pyr_ ratio (which is indicative of mitochondrial coupling when pyruvate is provided as an oxidative substrate) was significantly higher for the WT flies fed the SD compared to the WT flies fed the HSD and the MSD diets (*p* < 0.001 and *p* = 0.019; Figure 2A) as well as to the MPC1^def^ flies under the same diet (*p* < 0.001; Figure 2A). The E_max_/P_max_ ratios were very similar for each condition, with no significant differences among groups, denoting that the phosphorylation system is not limiting oxygen consumption in the different groups (Figure 2B). The different contribution of each substrate injected to stimulate the electron flux into the ETS and hence mitochondrial oxygen consumption was then calculated from the different respiration rates measured. The contribution of malate to the mitochondrial oxygen consumption was higher when the WT flies were fed the HSD compared to those fed the SD and the MSD (all *p*-values < 0.001; Figure 2C) as well as when both genotypes exposed to the HSD were compared (*p* < 0.001; Figure 2C). Interestingly, we observed that, for the SD and MSD experimental diets, the MPC1^def^ flies had significantly increased contribution of proline than the WT flies (*p* = 0.011 and *p* = 0.016; Figure 2D). The WT flies fed the HSD also relied more on proline as an oxidative substrate for respiration than WT flies fed the SD or MSD (*p* = 0.020 and *p* = 0.013), reaching the same contribution as the MPC1^def^ flies (increase of approximately 50%; Figure 2D). For the contribution of the other substrates (succinate and G3P), no significant differences were detected between the different groups (Figure 2E,F).

### 2.3. Metabolite Analysis

**Figure 3:**
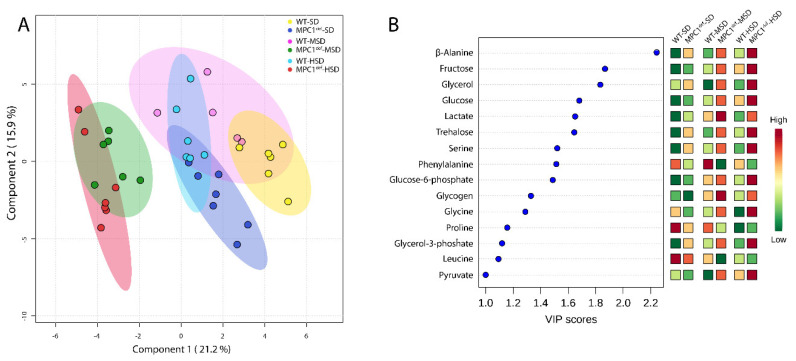
Metabolite profile analysis in *Drosophila melanogaster* WT and MPC1^def^ exposed to the different experimental diets (SD, MSD, or HSD): (**A**) Partial least squares discriminant analysis (PLS-DA) score plots of metabolites in WT and MPC1^def^ flies fed the different experiment diets. Ellipses represent 95% confidence intervals for each individual group on PLS-DA plots with the variance proportion represented by components 1 and 2. (**B**) Variable importance of projection (VIP) scores of PLS-DA component 1, which identify the key metabolites driving the metabolomic signature for WT and MPC1^def^ flies fed the different experimental diets (VIP > 1.0): N = 6–7 for each group.

Relative metabolite abundance was analyzed using the multivariate partial least squares discriminant analysis (PLS-DA) [27] to evaluate potential metabolic shifts due to impairment of mitochondrial pyruvate import and dietary sucrose (Figure 3A). The two main PLS components explained 21.2% and 15.9% of the total variance, respectively (Figure 3A). Both components displayed good estimates of the predictive ability of the model following cross-validation (*Q^2^* = 0.66 and *R^2^* = 0.77 for component 1; *Q^2^* = 0.79 and *R^2^* = 0.89 for component 2). In accordance with what we previously observed in WT and MPC1^def^ flies fed the SD at 15 days old [22], the WT and MPC1^def^ flies fed the SD separated on the first PLS component, suggesting that the genotypes could have a different metabolic signature (Figure 3A).

However, WT flies and MPC1^def^ flies fed the SD both clustered with WT flies fed the MSD and the HSD (Figure 3A). A clear separation was observed between this cluster and MPC1^def^ flies fed the MSD and the HSD on the first PLS component. This result indicates that MPC1^def^ flies fed the MSD and HSD have a different metabolic signature compared to the other groups (Figure 3A). Variable importance of projection (VIP) scores identified 15 metabolites significantly driving the specific metabolomic signature between groups (i.e., metabolites with VIP > 1; Figure 3B and Table 2). To further characterize the differences between groups, we then compared the levels of these 15 metabolites using two-way ANOVAs.

#### 2.3.1. Sugars and Glycolysis Intermediates

**Figure 4:**
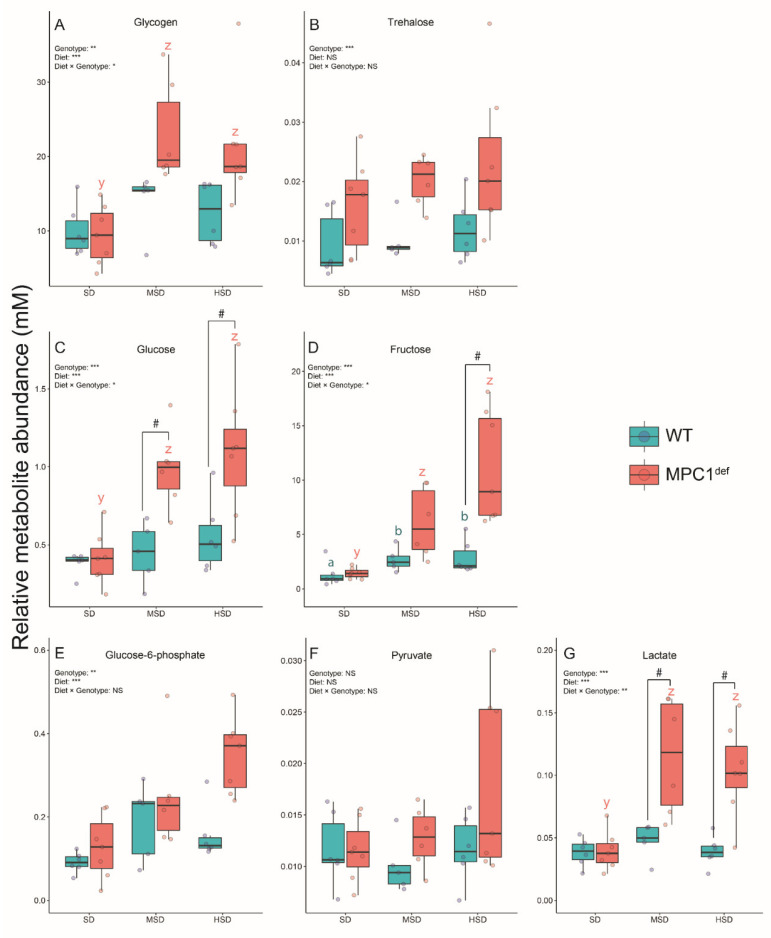
Sugar and glycolysis intermediate levels measured in *Drosophila melanogaster* WT and MPC1^def^ exposed to the different experimental diets (SD, MSD, or HSD): (**A**) glycogen levels, (**B**) trehalose levels, (**C**) glucose levels, (**D**) fructose levels, (**E**) glucose-6-phosphate levels, (**F**) pyruvate levels, and (**G**) lactate levels. Results correspond to the mean ± SEM for each condition (N = 6–7). Statistical analysis: stars represent statistical results from ANOVAs with * *p* < 0.05, ** *p* < 0.01, and *** *p* < 0.001; # represents significant differences between genotypes for each experimental diet detected using the estimated marginal means method; and letters denote differences between experimental diets for each genotype, with “a” and “b” representing differences between the WT flies and “y” and “z” representing differences between the MPC1^def^ flies.

Several carbohydrate-derived metabolites displayed significant VIP scores (Table 2). The common polysaccharide glycogen had significantly higher levels in the MPC1^def^ flies fed the MSD and the HSD compared to those fed the SD (*p* < 0.001 and *p* = 0.001 respectively; Figure 4A). Moreover, MPC1^def^ flies fed the MSD and HSD had generally but not significantly higher glycogen levels than WT flies fed the same diets. For trehalose, the most common circulating sugar in fly hemolymph, no genotype × diet interaction was detected (Figure 4B). However, a strong effect of genotype was observed with the MPC1^def^ flies tending to have higher concentrations of trehalose than WT flies (Table 2 and Figure 4B).

The concentration of glucose in the MPC1^def^ flies was significantly higher than in the WT flies on the MSD and the HSD (*p* = 0.016 and *p* = 0.007, respectively; Figure 4C). Moreover, the glucose concentration in the MPC1^def^ flies was significantly lower on the SD than on the MSD and the HSD (*p* = 0.004 and *p* < 0.001, respectively; Figure 4C). Surprisingly, levels of glucose in WT flies were similar across diets (Figure 4C). Fructose levels were also generally higher in MPC1^def^ flies compared to WT flies on the MSD and HSD, but this increase was only significant on the HSD (*p* < 0.001; Figure 4D). Moreover, fructose levels were significantly higher in the flies fed the HSD compared to the SD for both genotypes (*p* = 0.035 and *p* < 0.001 for WT and MPC1^def^ flies, respectively; Figure 4D) as well as in the MPC1^def^ flies fed the MSD compared to the SD (*p* < 0.001; Figure 4D).

Three metabolites from glycolysis were also involved in driving the differences among the groups. For glucose-6-phosphate, the genotype × diet interaction was not significant. However, single effects of genotype and diet were detected, with WT flies having generally lower levels of glucose-6-phosphate than MPC1^def^ flies, and slightly increasing concentrations of this metabolite were also detected with the increasing concentrations of dietary sucrose in both genotypes (Figure 4E). Surprisingly, pyruvate was not significantly different among groups (Figure 4F) with no effect of genotype, diet, or their interaction. Pyruvate can be converted to lactate in anaerobic conditions, and we found that the MPC1^def^ flies had higher lactate concentrations when fed the MSD and the HSD compared to the SD (*p*-values < 0.001; Figure 4G). Moreover, lactate levels in WT flies fed the MSD and HSD were significantly lower than in MPC1^def^ flies fed the same diets (*p* = 0.009 and *p* = 0.001, respectively; Figure 4G).

#### 2.3.2. Amino Acids

**Figure 5:**
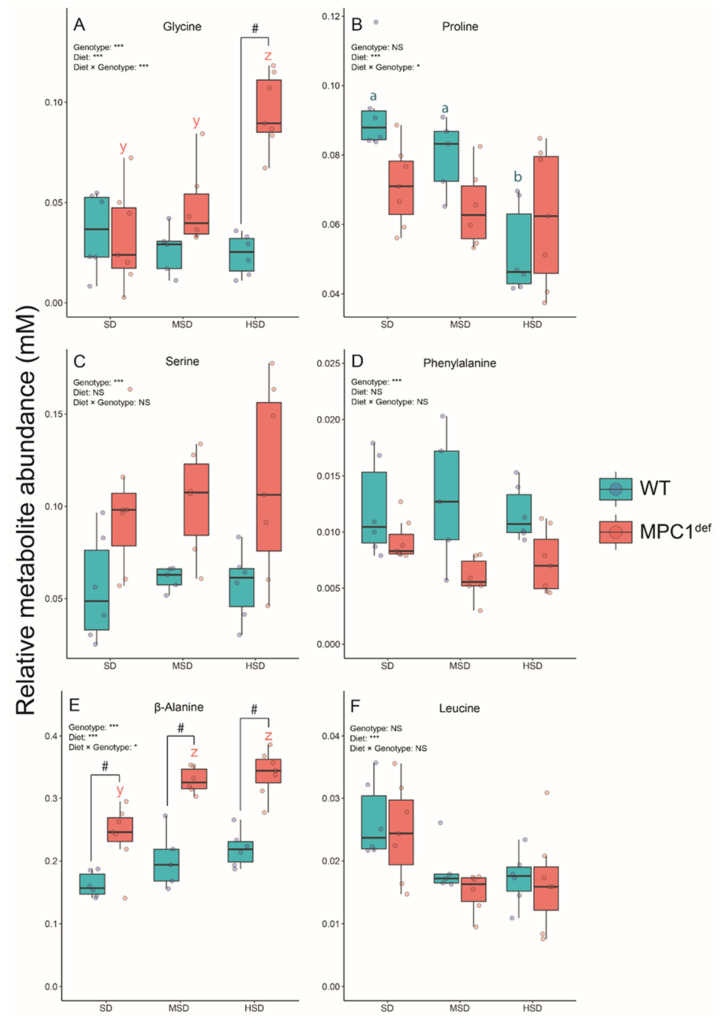
Amino acid levels measured in *Drosophila melanogaster* WT and MPC1^def^ exposed to the different experimental diets (SD, MSD, or HSD): (**A**) glycine levels, (**B**) proline levels, (**C**) serine levels, (**D**) phenylalanine levels, (**E**) β-alanine levels, and (**F**) leucine levels. Results correspond to the mean ± SEM for each condition (N = 6–7). Statistical analysis: stars represent statistical results from ANOVAs with * *p* < 0.05, and *** *p* < 0.001; # represents significant differences between genotypes for each experimental diet detected using the estimated marginal means method; and letters denote differences between experimental diets for each genotype, with “a” and “b” representing differences between the WT flies and “y” and “z” representing differences between the MPC1^def^ flies.

Six amino acids displayed significant VIP scores (Table 2). Glycine levels were higher in the MPC1^def^ flies compared to the WT flies when fed the HSD (*p* < 0.001; Figure 5A). Moreover, the MPC1^def^ flies fed the HSD had significantly higher levels of this metabolite than when fed the other diets (all *p*-values <0.001; Figure 5A). For proline, the WT flies fed the SD and MSD had significantly higher levels than those fed the HSD (*p* < 0.001 and *p* = 0.029, respectively; Figure 5B). However, the MPC1^def^ flies had similar levels of proline across diets and presented no significant differences compared to the WT flies (Figure 5B). No significant genotype × diet effects were detected for either serine or phenylalanine, but strong genotype effects were observed with generally higher levels of serine and lower levels of phenylalanine in MPC1^def^ flies (Figure 5C,D). For β-alanine, the concentrations were significantly higher for the MPC1^def^ flies compared to WT flies for each diet (*p* = 0.006, *p* < 0.0001, and *p* < 0.001 for SD, MSD, and HSD, respectively; Figure 5E). In the MPC1^def^ flies, the levels of β-alanine were similar between the MSD and the HSD, but both groups presented significantly higher levels than flies fed the SD (*p* = 0.001 and *p* < 0.001, respectively; Figure 5E). Finally, leucine levels were only influenced by the diet with slightly decreasing levels observed in flies from both genotypes fed the MSD and the HSD compared to the SD (Figure 5F).

#### 2.3.3. Polyols

**Figure 6:**
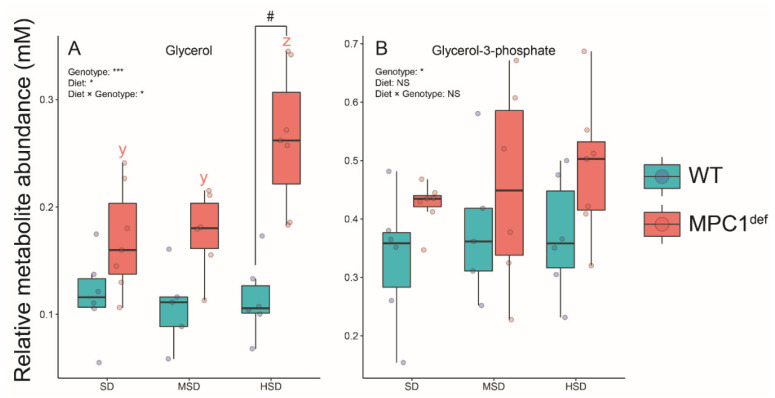
Polyol levels measured in *Drosophila melanogaster* WT and MPC1^def^ exposed to the different experimental diets (SD, MSD, or HSD): (**A**) glycerol levels and (**B**) glycerol-3-phosphate levels. Results correspond to the mean ± SEM for each condition (N = 6–7). Statistical analysis: stars represent statistical results from ANOVAs with * *p* < 0.05, and *** *p* < 0.001; # represents significant differences between genotypes for each experimental diet detected using the estimated marginal means method; and letters denote differences between experimental diets for each genotype, with “y” and “z” representing differences between the MPC1^def^ flies.

Lastly, two other metabolites (glycerol and glycerol-3-phosphate) also significantly drove the specific metabolomic signature between groups. Glycerol levels were higher in MPC1^def^ flies fed the HSD compared to those fed the SD and the MSD (*p* = 0.008 and *p* = 0.021, respectively; Figure 6A). Moreover, significant differences were detected between genotypes on the HSD, with higher glycerol levels detected in MPC1^def^ flies (*p* < 0.001; Figure 6A). The glycerol-3-phosphate levels were only influenced by the genotype, with slightly higher levels observed in the MPC1^def^ flies compared to the WT flies (Figure 6B).

## 3. Discussion

In this study, we aimed to evaluate the involvement of the MPC in the metabolism of fruit flies fed different experimental diets varying in sucrose concentrations to investigate the role of this protein in the occurrence of metabolic inflexibility. Our results showed that, independently of the experimental diets, MPC1^def^ flies had similar mitochondrial respiration rates, which were generally lower than WT flies. When exposed to the HSD, WT flies displayed decreased mitochondrial respiration rates compared to MPC1^def^ flies as well as slightly decreased *MPC1* gene expression. We also found an increased proline contribution in WT flies fed the HSD and in MPC1^def^ flies for all experimental diets, which was consistent with the levels of proline detected. These results suggest that proline is an important oxidative substrate that might partially alleviate mitochondrial dysfunctions in Drosophila when an impairment of carbohydrate metabolism such as a problem in pyruvate transport [22] or a metabolic inflexibility due to excess dietary carbohydrates occurs. Surprisingly, few metabolite levels were altered in WT flies fed the MSD or the HSD. On the contrary, MPC1^def^ flies fed the MSD and the HSD display significant accumulation of several important metabolites known to be involved in metabolic inflexibility such as glycogen, glucose, fructose, lactate, and glycerol. Overall, these results suggest that metabolic inflexibility starts to occur in WT flies after 15–25 days of exposure to the HSD, as seen with decreased mitochondrial respiration rates and *MPC1* expression, whereas contradictory to our hypothesis, the MPC1^def^ flies display metabolic inflexibility independently of the diet provided. Our study therefore indicates that proper functioning of the MPC is a prerequisite for maintaining metabolite levels and mitochondrial functions and is necessary for the maintenance of homeostasis during changes in diet macronutrients. Thus, this further highlights the role of the MPC as a crucial metabolic control point in Drosophila, especially considering the highly variable sugar concentration found in their diet in nature.

As expected, MPC1^def^ flies displayed significantly lower mitochondrial oxygen consumption than WT flies when pyruvate was used as the oxidative substrate (CI_pyr_-OXPHOS) and when flies were exposed to either the SD or the MSD. This is in accordance with our previous study showing decreased pyruvate-induced respiration rate in 15-day-old males fed the SD [22] as well as with other studies showing reduced pyruvate-stimulated respiration in yeast when the MPC1 was genetically deleted and in human cells with siRNA-mediated knock-down of either MPC1 or MPC2 [18,28,29]. However, respiration rates were generally significantly higher for the MPC1^def^ flies when both genotypes were fed the HSD. We then calculate the P_pyr_/L_pyr_ ratio, which is an indicator of coupling efficiency [30,31]. This ratio was significantly lower for the MPC1^def^ flies compared to the WT flies on the SD and similar between genotypes on either the MSD or the HSD. Thus, it indicates that WT flies fed the HSD exhibit similar dysfunction as the MPC1^def^ flies fed the other diets and is also consistent with the decreased *MPC1* gene expression observed in these flies, which might represent early metabolic inflexibility due to excess dietary carbohydrates. Indeed, it has been shown in Drosophila that high sugar diets generally lead to several metabolic disorders such as cardiomyopathy, obesity, and insulin resistance [32,33,34,35] as well as mitochondrial dysfunctions [36], which are all related to metabolic inflexibility [2]. With the addition of other oxidative substrates, we observed that, while succinate and G3P were not significantly different between the different groups, MPC1^def^ flies on all diets as well as WT flies fed the HSD had an increased capacity to oxidize proline (increase of approximately 45–50% for all groups) and that WT flies fed the HSD also had an increased capacity to oxidize malate (increase of approximately 11%). These increased contributions, especially for proline, might help alleviate mitochondrial dysfunctions due to either impairment of pyruvate transport (as previously demonstrated in [22]) or excess dietary carbohydrates. The ETS capacity is similar for all groups, thus suggesting that the ATP synthase is not limiting the mitochondrial respiration as seen with the E_max_/P_max_ ratio [30,31,37]. Altogether, these results suggest that the MPC1 deficiency as well as exposure to the HSD for the WT flies affect the capacity of mitochondria to oxidize substrates, which participates to the development of metabolic inflexibility.

Metabolite levels measured using NMR spectroscopy allowed us to further characterize the role of the MPC in the occurrence of metabolic inflexibility. For sugar levels, we found higher concentrations in the MPC1^def^ flies compared to the WT flies, and the difference between genotypes was even greater with the increased concentration of sucrose in the diet, not only for glucose and fructose but also for trehalose and glycogen. This is consistent with other studies [16,18] in which the same tendencies for glucose, glycogen, and trehalose were observed in transheterozygous MPC flies. Specifically, McCommis et al. [16] showed that Drosophila MPC1 null mutants exposed to a 18% sucrose diet have elevated blood sugar concentrations and glucose intolerance accompanied by impaired glucose-stimulated insulin secretion as well as decreased lifespan. This phenotype was associated with accumulation of glucose, trehalose, glycogen, pyruvate, and several sugar alcohols as well as decreased triglyceride content, suggesting that these mutants are unable to maintain carbohydrate and lipid homeostasis in response to changes in dietary sugar [16]. The discrepancies found between our study and McCommis et al. [16] (such as pyruvate levels) are likely attributable to the differences between the MPC1 null mutants which display a complete MPC1 knockout compared to our model that only has a mild MPC1 deficiency. Moreover, Bricker et al. [18] demonstrated a depletion of TCA cycle intermediates in transheterozygous MPC flies. Although TCA cycle intermediates were not found to significantly drive the differences between groups in our model, some metabolites such as malate, isocitrate, and citrate were lower in both genotypes fed either the MSD or the HSD (Table 2). Surprisingly, the WT flies had lower variations in sugar concentrations when exposed to the diets, suggesting that they can efficiently use and store these sugars. Only fructose was statistically elevated in WT flies fed the HSD compared to the other diets. These results suggest that the MPC1^def^ flies have impaired sugar metabolism, which translates into metabolic inflexibility as seen with the overall decreased mitochondrial respiration. However, the WT flies did not display this sugar accumulation on the HSD despite decreased mitochondrial respiration rates and *MPC1* gene expression. One explanation for these results might be that WT flies fed the HSD were sampled at the onset of metabolic inflexibility, with decreased mitochondrial respiration rates and decreased *MPC1* expression being early manifestations of metabolic inflexibility.

The levels of glucose-6-phosphate augmented in the MPC1^def^ flies with increased concentrations of sucrose in the diet and the WT flies had lower concentrations of glucose-6-phosphate than the MPC1^def^ flies on the HSD. This correlates with the glucose levels detected and further suggests that the MPC1^def^ flies have impaired sugar metabolism. For pyruvate, we predicted an accumulation in the MPC1^def^ flies, as already demonstrated in transheterozygous MPC flies [16,18]. Surprisingly, we did not find any significant differences between groups for pyruvate, although we observed slightly higher concentrations in the MPC1^def^ flies fed the HSD, which might be explained by different experimental designs (such as fly genotype, age, sex, and specific food composition). Pyruvate can also be converted to lactate in the cytosol. It has been shown that homozygous mutant mice expressing a truncated form of the MPC2 protein had elevated blood lactate, suggesting that a higher proportion of pyruvate was converted into lactate [38]. Moreover, impaired import of pyruvate via loss of the MPC has also been shown to enforce the Warburg effect in cancer cells [39]. Consistent with this, we found higher concentrations of lactate in the MPC1^def^ flies compared to the WT flies on the MSD and the HSD diets. For flies fed the SD, the concentrations in both genotypes were almost identical, and the WT flies fed the three experimental diets showed no differences between each other. This suggests that, in the MPC1^def^ flies, a proportion of the pyruvate generated through glycolysis might be converted to lactate rather than accumulated like previously hypothesized, suggesting a metabolic remodeling in the MPC1^def^ flies favoring anaerobic glycolysis when these flies are fed the MSD and the HSD.

The amino acids glycine and serine displayed elevated concentrations in the MPC1^def^ flies, especially when they were fed the HSD. This could be explained by the fact that these two metabolites can interconvert with glycolytic intermediates [40] and is consistent with the accumulation of glucose, fructose, and glucose-6-phosphate detected in MPC1^def^ flies fed the HSD. On the other hand, proline and phenylalanine which can interconvert with TCA cycle intermediates were generally lower in MPC1^def^ flies and in WT flies fed the HSD. This is consistent with Bricker et al. [18] as well as with the decreased mitochondrial respiration rates observed in MPC1^def^ flies and in WT flies fed the HSD, as the TCA cycle is the main provider of NADH and FADH_2_ that allow the electron transport into the ETS. Considering proline, it is likely that this amino acid is directly used as an electron donor to the ETS in Drosophila, as already demonstrated in several insects [8,41,42,43], and confirms its importance as an oxidative substrate when impaired carbohydrate metabolism occurs in Drosophila [22]. Surprisingly, β-alanine was the metabolite with the highest VIP score (Section 2.3.2; Table 2). In Drosophila, β-alanine has been shown to be important in determining cuticle melanization [44], signaling in photoreceptor synapse [45], and aggressive behavior [46,47,48]. It is however unclear whether and how the levels of this metabolite could be linked to the MPC or excess dietary sucrose. Nevertheless, β-alanine levels were significantly higher in MPC1^def^ flies fed the MSD and the HSD compared to MPC1^def^ flies fed the SD or to WT flies and were slightly increased in WT flies fed the MSD and the HSD compared to those fed the SD. Interestingly, an early study showed that injection of β-alanine in Drosophila caused inhibition of glucose catabolism [49], which aligns well with our results. However, we cannot infer about the specific relationship between this metabolite and the different results obtained here.

An accumulation of glycerol (and to a lesser extent of G3P) was also observed in MPC1^def^ flies, especially when they were fed the HSD. In Drosophila, glycerol and G3P are involved in tri-/diglyceride formation and degradation [50]. Thus, our results indicate that the impairment of carbohydrate metabolism is associated with accumulation of building blocks for fat reserves. This is consistent with other studies demonstrating that dietary supplementation of sugars in Drosophila results in these compounds being incorporated and stored as fat [34,51]. This indicates that the WT flies can efficiently convert dietary sucrose into other metabolites and that the MPC1^def^ flies display metabolic inflexibility resulting in glycerol accumulation.

In summary, our study shows that the MPC1^def^ flies have an impairment of pyruvate-stimulated respiration and accumulates more sugars, lactate, and glycerol than the WT flies, especially when fed the HSD, which is symptomatic of metabolic inflexibility. However, while the WT flies also displayed reduced pyruvate-stimulated respiration when fed the HSD, their metabolic profile was only slightly different from the WT flies fed the SD. Other studies in Drosophila have shown that diets high in carbohydrates may cause obesity, insulin resistance, and cardiomyopathy [32,33,34,35], which are often a result of metabolic inflexibility [2]. However, compared to these other studies which exposed eggs or larvae to high carbohydrate content diets, we exposed adult flies to excess dietary sucrose from 15 days old, the age at which all larval fat cells have been removed [52,53]. The only evidence of metabolic inflexibility that we observed in WT flies was the severe decreased mitochondrial respiration when they were fed the HSD, which coincided with a slight decrease of *MPC1* gene expression. It is possible that this phenotype represents an early stage of metabolic inflexibility and that prolonging exposure to the HSD would have resulted in accumulation of metabolites as observed in MPC1^def^ flies. Moreover, MPC1^def^ flies clearly display a loss of homeostasis due to metabolic inflexibility. Therefore, we can conclude that the MPC is an essential protein for Drosophila metabolism to maintain proper metabolic homeostasis during changes in dietary resources. Further studies evaluating the role of this protein during other environmental challenges such as caloric restriction and changes in temperatures could bring new understanding about the importance of MPC in the intermediary metabolism of Drosophila.

## 4. Materials and Methods

### 4.1. Drosophila Maintenance and Experimental Design

Drosophila melanogaster genotypes w^1118^ and w[1118]; P{w[+ mC] = XP}Mpc1[d00809], hereafter referred as WT and MPC1^def^, respectively, were obtained from the Bloomington Drosophila Stock Center (Bloomington, IN, USA). The MPC1^def^ genotype was generated using P-element insertion, which frequently interferes directly with gene function [23,24,25] and was previously characterized by our group at the mitochondrial level (see [22]). While it is possible that the P-element insertion in MPC1^def^ flies caused other unknown mutations, the effects of the MPC1 deficiency detected on mitochondrial metabolism indicate that the main effects observed on the phenotype are due to this specific deficiency. Specifically, MPC1^def^ male flies 15 days old have a 2-fold decrease in *MPC1* gene expression and have reduced pyruvate-induced mitochondrial oxygen consumption compared to WT male flies of the same age [22]. The flies from both genotypes were maintained in an incubator at 24 **°**C and 50% humidity with a 12:12 h light–dark cycle. Drosophila were fed a standard diet (SD), which consisted of sugar 5 g·L^−1^, agar 6 g·L^−1^, yeast 27 g·L^−1^, and cornmeal 53 g·L^−1^ mixed in 1 L of tap water and complemented with methyl-p-hydroxybenzoate dissolved in 95% ethanol (10% *w*/*v*) to prevent mold growth and with 0.4% (*v*/*v*) propionic acid to prevent mite contamination. Male flies were collected every day as they hatched and were transferred to fresh vials of SD at a density of 15 flies/vial. When the flies reached 15 days old, they were either kept on SD or transferred on two different experimental diets consisting of SD complemented with either 18% (*w*/*v*) sucrose (MSD) or 30% (*w*/*v*) sucrose (HSD). The flies were exposed to each diet for 15–25 days prior to being collected at 30–40 days old. Mitochondrial respiration was immediately performed on freshly dissected thorax. For NMR metabolite extraction and quantification, whole flies were rinsed in phosphate buffer saline (PBS), flash frozen in liquid nitrogen, and kept at −80 **°**C until extraction of hydrophilic metabolites.

### 4.2. MPC1 Gene Expression

*MPC1* gene expression was evaluated as previously described [22]. Briefly, total RNA extraction was performed on whole flies (N = 5 for each genotype fed with each diet) using TRIzol reagent (Millipore-Sigma, Oakville, ON, Canada) according to the manufacturer’s protocol, and the 260/280 nm and 260/230 nm absorbance ratios were used to verify the quality of the RNA in each sample. Total RNA (1 µg) was reverse transcribed using the SensiFAST™ cDNA synthesis kit (Bioline, London, UK). Real-time quantitative PCR was performed on a CFX Connect^TM^ (Biorad, Mississauga, ON, Canada) by incubating the cDNA with forward and reverse primers for *MPC1* (F: 5′-CTCAAAGGAGTGGCGGGATT-3′; R: 5′-CAGGGTCAGAGCCAATGTCA-3′) and *rps20* (F: 5′-GCATCACCACCCGTAAGA-3′; R: 5′-GTGGATTCTCATCTGGAAGCG-3′) and by using the SensiFAST™ SYBR^®^ No-ROX kit (Bioline, London, UK) using the following protocol: denaturation for 2 min at 95 °C, followed by 34 cycles of 5 s at 95 °C and 30 s at 55 °C. Results were analyzed with the ΔΔCq method with *rps20* as the reference gene.

### 4.3. Mitochondrial Respiration

Mitochondrial respiration was measured on permeabilized thorax as previously described [26,54]. The thoraxes of 3 flies were collected, dissected, and mechanically and chemically permeabilized using sharp forceps and a solution of saponin (62.5 μg·mL^−1^) in BIOPS medium consisting of 10 mmol.L^−1^ Ca-EGTA buffer, 0.1 μM free Ca^2+^, 20 mmol·L^−1^ imidazole, 20 mmol·L^−1^ taurine, 50 mmol·L^−1^ K-MES, 0.5 mmol·L^−1^ dithiothreitol, 6.56 mmol·L^−1^ MgCl_2_, 5.77 mmol·L^−1^ ATP, 15 mmol·L^−1^ phosphocreatine, and pH 7.1 [55,56]. The permeabilized fibers were then transferred to the chambers of an Oxygraph O2K (Oroboros instruments, Innsbruck, Austria). For each group (each genotype and each diet), 6 different pools of 3 permeabilized thoraxes were evaluated (N = 6). Mitochondrial respiration was measured following the addition of different substrates and inhibitors. First, the LEAK respiration with pyruvate (10 mM) at the level of complex I was measured (CI_pyr_-LEAK). Addition of ADP (5 mM) allowed the measurement of mitochondrial oxygen consumption when the transport of electrons from complex I was coupled to the phosphorylation of ADP to ATP (CI_pyr_-OXPHOS). These respiration rates were used to calculate the P_pyr_/L_pyr_ ratio (CI_pyr_-OXPHOS/CI_pyr_-LEAK), which is a good proxy for mitochondrial quality and mitochondrial coupling [31]. Malate (2 mM) was then injected to maintain the carbon flux into the TCA cycle (CI_pyr+mal_-OXPHOS), followed by addition of cytochrome *c* (15 μM, CIc-OXPHOS) to determine the intactness of the outer mitochondrial membrane [26,57]. Addition of cytochrome c did not increase mitochondrial oxygen consumption in all preparations tested, attesting that the outer mitochondrial membrane was intact (results not shown). Several other substrates were then injected: proline (5 mM), which provides electrons to the ETS via the proline dehydrogenase (CI+ProDH-OXPHOS); succinate (20 mM), which brings electrons to the ETS through complex II (CI+ProDH+CII-OXPHOS); and G3P (15 mM), that allows the transport of electrons to the ETS via the mG3PDH (CI+ProDH+CII+mG3PDH-OXPHOS). The respiration rates measured with these different substrates were used to evaluate the contribution of each substrate to mitochondrial oxygen consumption during OXPHOS: (i) malate contribution = (CI_pyr+mal_-OXPHOS − CI_pyr_-OXPHOS)/CI_pyr_-OXPHOS; (ii) proline contribution = (CI+ProDH-OXPHOS − CIc-OXPHOS)/CIc-OXPHOS; (iii) succinate contribution = (CI+ProDH+CII-OXPHOS − CI+ProDH-OXPHOS)/CI+ProDH-OXPHOS; and (iv) G3P contribution = (CI+ProDH+CII+mG3PDH-OXPHOS − CI+ProDH+CII-OXPHOS)/CI+ProDH+CII-OXPHOS). Injection of carbonyl cyanide 4-(trifluoromethoxy)phenylhydrazone (FCCP, steps of 0.5–1 μM) was then performed to measure the non-coupled respiration, i.e., the non-phosphorylating respiration stimulated to maximal oxygen consumption (CI+ProDH+CII+mG3PDH-ETS), and to calculate the E_max_/P_max_ ratio (CI+ProDH+CII+mG3PDH-ETS/CI+ProDH+CII+mG3PDH-OXPHOS), which indicates a possible limitation of the phosphorylation system if higher than 1.0 [31,58]. Inhibition of complexes I, II, and III by rotenone (0.5 μM), malonate (5 mM), and antimycin A (2.5 μM), respectively, were then performed to evaluate the residual oxygen consumption, which was subtracted from the previous respiration rates measured. All measurements were performed at 24 °C and are presented as means of mass-specific respiration rates expressed as pmol O_2_·s^−1^·mg^−1^ of permeabilized fibers ± s.e.m.

### 4.4. Metabolite Extraction and Analysis

To obtain a profile of key metabolites, we first extracted hydrophilic metabolites by homogenizing 21 flies in a 1:1 acetonitrile–distilled water solution using a pellet pestle (2 × 40 up and down) on ice. The homogenates were then centrifuged at 13,500 rpm for 10 min at 4 **°**C. The supernatant was collected and transferred to a glass test tube and frozen at −80 **°**C for at least 24 h. For the NMR analysis, the samples were thawed at room temperature and evaporated under nitrogen in a lightly heated water bath. The internal standard 4,4-dimethyl-4-silapentane-1-sulfonic acid (DSS) was then added to the sample (0.5 mM of DSS final concentration) and the resulting mix was solubilized in 700 μL of deuterium oxide. The ^1^H-NMR spectra for the extracted metabolites, dissolved in H_2_O–D_2_O (9:1) were recorded on a Bruker Advance III 400 MHz spectrometer at 298 K equipped with a 5-mm TCI CryoProbe. The noesypr1d pulse sequence was used. A total of 128 scans of 64,000 data points were recorded with a recycle delay of 1 s per scan. The spectral width was set at 12 KHz, and the acquisition time was set at 6.6 s. The obtained spectra (N = 5–7 for each genotype) were analyzed using the Chenomx NMR Suite (Chenomx Inc., Edmonton, Canada) and the Human Metabolome DataBase (HMDB) [59]. A series of 49 hydrophilic metabolites were identified and included in the analysis.

### 4.5. Statistical Analysis

Statistical analyses were performed with R software (version 3.1.0, Free Software Foundation, Boston, MA, USA) and MetaboAnalyst 4.0 [27]. For *MPC1* gene expression, mitochondrial oxygen consumption, mitochondrial ratios, and relative metabolite abundance, the data were fitted to a linear model and were analyzed using a two-way ANOVA with the genotypes (WT and MPC1^def^) and the experimental diets (SD, MSD, and HSD) as fixed factors. If an interaction effect (genotype × diet) was detected, multiple comparisons were then tested with pairwise comparisons of the estimated marginal means using adjusted *p*-values (Tukey method). For these ANOVAs, normality was verified with the Shapiro–Wilk’s test, homogeneity of variances was verified using the Levene’s test, and data were transformed when required. The relative metabolite abundance was further analyzed using a partial least squares discriminant analysis (PLS-DA) in MetaboAnalyst 4.0 after sample normalization with DSS and after data log transformation and auto scaling (i.e., mean-centered and divided by the standard deviation of each variable) in order to minimize possible differences in concentration between samples. PLS-DA identified the metabolites driving the separation and/or clustering among genotypes by ascribing a variable importance of projection score (VIP). Variables with a VIP score of > 1.0 were considered important in the PLS-DA model.

## Figures and Tables

**Table 1:** *MPC1* relative gene expression measured in the wild-type (WT) and MPC1^def^ genotypes of *Drosophila melanogaster* exposed to the different diets (Standard Diet (SD), Medium-Sucrose Diet (MSD), or High-Sucrose Diet (HSD)): N = 5 for each genotype fed with each diet. Relative gene expression was calculated with the ΔΔCq method with *rps20* as the reference gene.

Group Used for Normalization	SD		MSD		HSD	
	WT	MPC1^def^	WT	MPC1^def^	WT	MPC1^def^
WT flies exposed to the SD	1.00 ± 0.04	0.62 ± 0.03	0.79 ± 0.03	0.52 ± 0.01	0.70 ± 0.03	0.50 ± 0.01
WT flies exposed to each diet	1.00 ± 0.04	0.62 ± 0.03	1.00 ± 0.01	0.65 ± 0.01	1.00 ± 0.01	0.78 ± 0.01

**Table 2:** Relative metabolites abundance and ascribed VIP scores assigned with PLS-DA on component 1 identifying the metabolites driving the separation and/or clustering among *Drosophila melanogaster* WT and MPC1^def^ exposed to the experimental diets (SD, MSD, or HSD).

Metabolites	Relative Abundance (mM)						
	Standard Diet (SD)		Medium Sucrose Diet (MSD)		High Sucrose Diet (HSD)		VIP Score Component 1
	WT	MPC1^def^	WT	MPC1^def^	WT	MPC1^def^	
β-Alanine	0.162 ± 0.008	0.240 ± 0.019	0.202 ± 0.021	0.329 ± 0.008	0.219 ± 0.012	0.340 ± 0.014	2.257
3-Hydroxybutyrate	0.228 ± 0.017	0.249 ± 0.045	0.183 ± 0.021	0.166 ± 0.026	0.177 ± 0.024	0.196 ± 0.027	0.450
3-Phosphoglycerate	0.969 ± 0.124	1.060 ± 0.176	1.008 ± 0.143	0.773 ± 0.087	1.097 ± 0.097	1.250 ± 0.249	0.051
Acetate	0.079 ± 0.013	0.064 ± 0.012	0.080 ± 0.011	0.094 ± 0.024	0.058 ± 0.006	0.052 ± 0.013	0.694
Acetoacetate	0.028 ± 0.003	0.022 ± 0.003	0.019 ± 0.002	0.015 ± 0.002	0.014 ± 0.003	0.024 ± 0.007	0.479
Alanine	0.182 ± 0.025	0.196 ± 0.016	0.193 ± 0.021	0.230 ± 0.019	0.170 ± 0.008	0.204 ± 0.015	0.794
Argininosuccinate	0.309 ± 0.077	0.275 ± 0.063	0.193 ± 0.048	0.361 ± 0.042	0.161 ± 0.029	0.193 ± 0.036	0.090
Asparagine	0.046 ± 0.005	0.053 ± 0.009	0.029 ± 0.005	0.033 ± 0.006	0.029 ± 0.004	0.039 ± 0.005	0.009
Carnitine	0.028 ± 0.003	0.018 ± 0.002	0.023 ± 0.003	0.018 ± 0.002	0.0200 ± 0.003	0.023 ± 0.004	0.629
Citrate	0.016 ± 0.002	0.017 ± 0.002	0.006 ± 0.002	0.007 ± 0.001	0.007 ± 0.002	0.008 ± 0.001	0.330
Citrulline	0.062 ± 0.005	0.090 ± 0.012	0.051 ± 0.007	0.072 ± 0.010	0.060 ± 0.008	0.063 ± 0.009	0.410
Aspartate	0.030 ± 0.004	0.027 ± 0.006	0.011 ± 0.002	0.010 ± 0.001	0.017 ± 0.002	0.016 ± 0.002	0.683
Fructose	1.316 ± 0.448	1.459 ± 0.185	2.697 ± 0.484	6.073 ± 1.303	2.900 ± 0.614	11.159 ± 1.938	1.879
Glutamine	0.542 ± 0.070	0.652 ± 0.079	0.413 ± 0.064	0.492 ± 0.051	0.560 ± 0.075	0.696 ± 0.094	0.664
Fructose 6-phosphate	0.103 ± 0.021	0.123 ± 0.023	0.117 ± 0.012	0.104 ± 0.025	0.116 ± 0.023	0.099 ± 0.033	0.311
Fumarate	0.005 ± 0.002	0.005 ± 0.001	0.006 ± 0.002	0.004 ± 0.001	0.004 ± 0.001	0.005 ± 0.001	0.199
Glucosamine 6-phosphate	0.054 ± 0.012	0.056 ± 0.009	0.112 ± 0.036	0.042 ± 0.003	0.085 ± 0.030	0.052 ± 0.022	0.854
Glucose	0.384 ± 0.027	0.411 ± 0.065	0.446 ± 0.087	0.981 ± 0.103	0.555 ± 0.094	1.096 ± 0.158	1.691
Glucose-6-phosphate	0.091 ± 0.010	0.128 ± 0.029	0.189 ± 0.041	0.249 ± 0.051	0.157 ± 0.026	0.349 ± 0.035	1.497
Glutamate	0.151 ± 0.013	0.131 ± 0.015	0.125 ± 0.014	0.085 ± 0.009	0.119 ± 0.018	0.122 ± 0.016	0.833
Glycerol	0.117 ± 0.016	0.170 ± 0.019	0.107 ± 0.017	0.176 ± 0.015	0.114 ± 0.014	0.264 ± 0.025	1.845
Glycerol 3-phosphate	0.332 ± 0.046	0.425 ± 0.014	0.384 ± 0.056	0.455 ± 0.070	0.371 ± 0.042	0.486 ± 0.045	1.126
Glycine	0.035 ± 0.008	0.033 ± 0.009	0.026 ± 0.005	0.048 ± 0.008	0.024 ± 0.004	0.095 ± 0.007	1.296
Glycogen	10.040 ± 1.393	9.447 ± 1.498	14.016 ± 1.825	23.099 ± 2.785	12.449 ± 1.687	21.292 ± 2.958	1.337
Isocitrate	0.045 ± 0.006	0.055 ± 0.008	0.028 ± 0.003	0.039 ± 0.004	0.035 ± 0.007	0.037 ± 0.005	0.061
Isoleucine	0.013 ± 0.002	0.015 ± 0.001	0.009 ± 0.001	0.010 ± 0.002	0.009 ± 0.001	0.008 ± 0.002	0.615
Lactate	0.038 ± 0.005	0.040 ± 0.006	0.048 ± 0.006	0.115 ± 0.019	0.039 ± 0.005	0.104 ± 0.014	1.661
Arginine	0.034 ± 0.004	0.035 ± 0.005	0.027 ± 0.006	0.027 ± 0.002	0.024 ± 0.005	0.021 ± 0.006	0.636
Cysteine	0.105 ± 0.027	0.085 ± 0.017	0.075 ± 0.024	0.063 ± 0.008	0.055 ± 0.009	0.098 ± 0.030	0.032
Leucine	0.026 ± 0.002	0.025 ± 0.003	0.019 ± 0.002	0.015 ± 0.001	0.017 ± 0.002	0.017 ± 0.003	1.010
Proline	0.093 ± 0.005	0.071 ± 0.004	0.080 ± 0.005	0.065 ± 0.005	0.052 ± 0.005	0.062 ± 0.007	1.1642
Lysine	0.076 ± 0.010	0.054 ± 0.007	0.025 ± 0.005	0.026 ± 0.002	0.020 ± 0.004	0.027 ± 0.005	0.815
Malate	0.097 ± 0.010	0.114 ± 0.014	0.019 ± 0.005	0.038 ± 0.009	0.052 ± 0.007	0.049 ± 0.010	0.309
Ornithine	0.041 ± 0.003	0.029 ± 0.004	0.025 ± 0.003	0.023 ± 0.002	0.017 ± 0.002	0.024 ± 0.002	0.888
Oxaloacetate	0.929 ± 0.140	1.079 ± 0.181	1.524 ± 0.233	1.531 ± 0.136	1.797 ± 0.049	1.793 ± 0.179	0.986
Phenylalanine	0.012 ± 0.002	0.009 ± 0.001	0.013 ± 0.003	0.006 ± 0.001	0.012 ± 0.001	0.007 ± 0.001	1.523
Phosphoenolpyruvate	0.055 ± 0.008	0.040 ± 0.007	0.039 ± 0.006	0.037 ± 0.002	0.041 ± 0.010	0.040 ± 0.007	0.534
Pyruvate	0.012 ± 0.001	0.012 ± 0.001	0.010 ± 0.001	0.013 ± 0.001	0.012 ± 0.001	0.018 ± 0.003	1.006
Serine	0.055 ± 0.012	0.099 ± 0.014	0.061 ± 0.003	0.102 ± 0.012	0.057 ± 0.008	0.113 ± 0.019	1.530
Succinate	0.091 ± 0.006	0.082 ± 0.011	0.085 ± 0.006	0.085 ± 0.006	0.053 ± 0.002	0.070 ± 0.003	0.428
Sucrose	0.015 ± 0.003	0.024 ± 0.003	0.028 ± 0.003	0.030 ± 0.004	0.024 ± 0.004	0.030 ± 0.006	0.965
Taurine	0.229 ± 0.020	0.245 ± 0.031	0.223 ± 0.027	0.268 ± 0.026	0.221 ± 0.020	0.279 ± 0.037	0.645
Threonine	0.086 ± 0.020	0.093 ± 0.012	0.054 ± 0.008	0.038 ± 0.003	0.050 ± 0.006	0.047 ± 0.008	0.793
Trehalose	0.009 ± 0.002	0.016 ± 0.003	0.010 ± 0.002	0.020 ± 0.002	0.012 ± 0.002	0.023 ± 0.005	1.654
Tyrosine	0.011 ± 0.001	0.015 ± 0.002	0.011 ± 0.001	0.008 ± 0.001	0.009 ± 0.001	0.008 ± 0.001	0.745
UDP-glucose	0.024 ± 0.005	0.025 ± 0.004	0.025 ± 0.006	0.016 ± 0.002	0.021 ± 0.003	0.023 ± 0.004	0.335
Valine	0.011 ± 0.002	0.010 ± 0.002	0.006 ± 0.001	0.008 ± 0.001	0.007 ± 0.001	0.007 ± 0.001	0.544

## Supplementary Materials

The following are available online at https://www.mdpi.com/2218-1989/10/10/411/s1, Table S1: Analyses of variances showing F values for mitochondrial respiration rates and mitochondrial ratios measured in *Drosophila melanogaster* WT and MPC1def exposed to the experimental diets (SD, MSD or HSD).
