# Supplementary material for: Role of the Mitochondrial Pyruvate Carrier in the Occurrence of Metabolic Inflexibility in Drosophila melanogaster Exposed to Dietary Sucrose
Source: Metabolites. 2020 Oct 14;10(10):411. doi: 10.3390/metabo10100411 (PMC7602203; doi:10.3390/metabo10100411)
Supplement: Supplementary file 1 [file metabolites-10-00411-s001.pdf]

Role of the mitochondrial pyruvate carrier in the occurrence of metabolic inflexibility in *Drosophila melanogaster* exposed to dietary sucrose.

Chloé J Simard[1], Mohamed Touaibia[1], Eric Pierre Allain[2], Etienne Hebert-Chatelain[3,4,5], Nicolas Pichaud[1,3*]

[1]Department of Chemistry and Biochemistry, Université de Moncton, Moncton, NB, Canada, E1 A 3E9.

[2]Atlantic Cancer Research Institute (ACRI), Moncton, NB, Canada, E1C 8X3.

[3]New Brunswick Centre for Precision Medicine (NBCPM), Moncton, NB, Canada, E1 A 3E9.

[4]Department of Biology, Université de Moncton, Moncton, NB, Canada, E1 A 3E9.

[5]Canada Research Chair in Mitochondrial Signaling and Physiopathology, Moncton, NB, Canada, E1A 3E9.

* Corresponding author: nicolas.pichaud@umoncton.ca

Supplementary material

Table S1. Analyses of variances showing F values for mitochondrial respiration rates and mitochondrial ratios measured in *Drosophila melanogaster* WT and $MPC1^{def}$ exposed to the experimental diets (SD, MSD or HSD).

**Mitochondrial respiration rates**	**DF**	**Genotype (df=1)**	**Diet (df=2)**	**Genotype*Diet (df=2)**
CI_{pyr}-LEAK	29	2.22	1.13	10.46***
CI_{pyr}-OXPHOS	29	5.22*	11.93***	15.69***
$CI_{pyr+mal}$-OXPHOS	30	5.34*	9.83***	16.19***
CI+proDH-OXPHOS	30	0.53	7.88**	13.74***
CI+proDH+CII-OXPHOS	30	0.74	8.18**	12.82***
CI+ProDH+CII+mG3P-OXPHOS	30	1.49	5.51**	16.52***
CI+ProDH+CII+mG3P-ETS	30	4.82*	2.87	15.74***
**Mitochondrial ratios**				
P_{pyr}/L_{pyr}	29	10.52**	8.06**	8.44**
E_{max}/P_{max}	30	3.94	1.65	0.24
Malate contribution	29	13.29*	8.29**	10.68***
Proline contribution	30	18.93***	4.83*	3.54*
Succinate contribution	30	1.98	0.25	2.66
G3P contribution	30	0.97	2.64	0.35

*P<0.05, **P<0.01, ***P<0.001; DF : denominator degree of freedom; df : numerator degree of freedom.
